# DNA Barcoding for Efficient Species- and Pathovar-Level Identification of the Quarantine Plant Pathogen *Xanthomonas*

**DOI:** 10.1371/journal.pone.0165995

**Published:** 2016-11-18

**Authors:** Qian Tian, Wenjun Zhao, Songyu Lu, Shuifang Zhu, Shidong Li

**Affiliations:** 1 Institute of Plant Protection, Chinese Academy of Agricultural Sciences, Beijing, China; 2 Institute of Plant Quarantine Research, Chinese Academy of Inspection and Quarantine, Beijing, China; 3 College of Plant Protection, Nanjing Agricultural University, Nanjing, China; Chinese Academy of Medical Sciences and Peking Union Medical College, CHINA

## Abstract

Genus *Xanthomonas* comprises many economically important plant pathogens that affect a wide range of hosts. Indeed, fourteen *Xanthomonas* species/pathovars have been regarded as official quarantine bacteria for imports in China. To date, however, a rapid and accurate method capable of identifying all of the quarantine species/pathovars has yet to be developed. In this study, we therefore evaluated the capacity of DNA barcoding as a digital identification method for discriminating quarantine species/pathovars of *Xanthomonas*. For these analyses, 327 isolates, representing 45 *Xanthomonas* species/pathovars, as well as five additional species/pathovars from GenBank (50 species/pathovars total), were utilized to test the efficacy of four DNA barcode candidate genes (16S rRNA gene, *cpn60*, *gyrB*, and *avrBs2*). Of these candidate genes, *cpn60* displayed the highest rate of PCR amplification and sequencing success. The tree-building (Neighbor-joining), ‘best close match’, and barcode gap methods were subsequently employed to assess the species- and pathovar-level resolution of each gene. Notably, all isolates of each quarantine species/pathovars formed a monophyletic group in the neighbor-joining tree constructed using the *cpn60* sequences. Moreover, *cpn60* also demonstrated the most satisfactory results in both barcoding gap analysis and the ‘best close match’ test. Thus, compared with the other markers tested, *cpn60* proved to be a powerful DNA barcode, providing a reliable and effective means for the species- and pathovar-level identification of the quarantine plant pathogen *Xanthomonas*.

## Introduction

The bacterial genus *Xanthomonas* comprises a large number of plant pathogens that are responsible for diseases of many economically important crops, including citrus, cassava, mangos, bananas, rice, wheat, sugarcane, beans, cruciferous vegetables, and many others. Notably, of the known species/pathovars of *Xanthomonas*, 14 are considered quarantine bacteria in China: *X*. *albilineans*, *X*. *arboricola* pv. *celebensis*, *X*. *axonopodis* pv. *betlicola*, *X*. *axonopodis* pv. *citri*, *X*. *axonopodis* pv. *manihotis*, *X*. *axonopodis* pv. *vasculorum*, *X*. *campestris* pv. *mangiferaeindicae*, *X*. *campestris* pv. *musacearum*, *X*. *cassavae*, *X*. *fragariae*, *X*. *hyacinthi*, *X*. *oryzae* pv. *oryzae*, *X*. *oryzae* pv. *oryzicola*, and *X*. *populi*. These quarantine species comprise a serious threat to the production of fruits and other commercially important food crops, as they are highly destructive to their respective host plants. Moreover, several of the aforementioned species/pathovars have been included in the European and Mediterranean Plant Protection Organization (EPPO) Pest Lists or are regarded as quarantine pests on a global scale. For these reasons, they are rigorously monitored by quarantine authorities, although the complexity of the genus makes it difficult to resolve this genus at the species or pathovar level. Indeed, while several methods, including isolation on semi-selective media, serology, and PCR-based methods, have been developed to identify these pathogens, the majority of these approaches are capable of identifying only one or two species/pathovars, and therefore do not have much versatility. As such, the objective of this study was to establish a new method for the efficient identification of all quarantine species and pathovars of *Xanthomonas*.

DNA barcoding is a molecular technique that was developed as a rapid and practical method for identifying organisms at the species level using specific DNA sequences [1]. Indeed, this technique has already been utilized for the molecular identification of multiple animal and plant species [2,3,4,5,6]. Currently, the identification of bacterial species is most commonly achieved via sequencing of the 16S rRNA gene, which encodes one of the structural RNA molecules in the small ribosomal subunit. Despite the positive features that have led to its preference as the barcode gene for bacteria, 16S rRNA gene sequencing often fails to provide sufficient information for species-level identification [7]. Therefore, several other candidate markers have been proposed, including DNA gyrase subunit B (*gyrB*), RNA polymerase genes (*rpoB*, *rpoD*, etc.), heat shock protein-related genes (*cpn60*, *hsp70*, etc.), and the bacterial DNA recombination protein gene *recA*, as have various combinations of markers. An ideal gene locus that meets the barcode criteria and is suitable for the identification and differentiation of microorganisms would be a powerful tool for differentiation of bacterial species/pathovars.

Genus *Xanthomonas* has been the subject of numerous taxonomical and phylogenetic studies, and the species-level classification of these organisms has been based largely on DNA–DNA hybridization, fatty acid profiling, repetitive element palindromic (rep)-PCR, and sequencing analysis of the 16S rRNA, *dnaK*, *fyuA*, *gyrB*, and *rpoD* genes, etc. [8,9,10,11]. However, species- and pathovar-level identification of *Xanthomonas* remains a significant challenge for diagnostic laboratories. The term pathovar is used to refer to a strain or set of strains with the same or similar characteristics, differentiated at infrasubspecific level from other strains of the same species or subspecies on the basis of distinctive pathogenicity to one or more plant hosts. Usually, pathovars are distinguished in terms of proved differences in host range [12]. However, Hajri *et al*. (2009) [13] found that there is a correlation between the composition of type III secretion system effector (T3E) repertoires and pathovars of *X*. *axonopodis*. Therefore, ‘Developing DNA barcode identification for Q-organisms Quarantine’ (QBOL, http://www.qbol.org/en/qbol.htm) has recommended that a ribosomal RNA gene (16S rRNA gene), a DNA gyrase gene (*gyrB*), and a T3E gene (*avrBs2*) be utilized as core barcoding genes for *Xanthomonas*. While *cpn60*, which encodes the 60 kDa chaperonin protein of bacteria and eukaryotes, is also considered an effective barcode gene for bacteria [14], there is little information to indicate whether this gene is suitable for barcoding of *Xanthomonas*.

Therefore, in this study, we investigated the DNA barcoding of quarantine species and pathovars of *Xanthomonas* in the context of biosecurity. Here, we selected the 16S rRNA gene, *gyrB*, *avrBs2*, and *cpn60* as candidate barcode genes. The primary objectives of the study were (1) to determine the universality of the primers used and quantify their amplification and sequencing success rates, and (2) to test the effectiveness of these DNA barcode candidates for species- and pathovar-level identification. The results of these analyses could provide a new digital identification method for the quarantine plant pathogen *Xanthomonas*.

## Materials and Methods

### Bacterial strains

All *Xanthomonas* strains used in this study were obtained from international culture collections [including the American Type Culture Collection (ATCC); National Collection of Plant Pathogenic Bacteria (NCPPB); Deutsche Sammlung von Mikroorganismen und Zellkulturen (DSMZ); International Collection Of Micro-organisms (ICMP); and Laboratory of Microbiology Gent Bacteria Collection (BCCM/LMG)], or were provided by research scientists from universities and research institutes in China (S1 Table). In total, 327 strains, comprising 45 species or pathovars and representing the majority of important *Xanthomonas* species, were collected for use in this study. All strains were routinely cultivated in nutrient broth (NB) at 28°C.

### DNA extraction, amplification, and sequencing

DNA was prepared from suspensions of freshly grown *Xanthomonas* cultures by boiling in a water bath for 10 min, followed by rapid cooling on ice for 5 min. After cooling, the bacterial lysates were centrifuged at 8,000 × *g* for 2–3 min and the DNA-containing supernatants were transferred to new centrifuge tubes and frozen at -20°C prior to use.

The following DNA regions were amplified for use as barcodes: 16S rRNA gene, amplified with primer pair *16sF*/*16sR* (designed in our laboratory); *cpn60*, amplified with primer pair *H1594*/*H1595* [15]; *gyr*B, amplified with primer pair *gyr-f*/*gyr-r* [16]; and *avrBs2*, amplified with primer pair *AvrBs2-F*/*AvrBs2-R* [13]. The *gyrB* and *avrBs2* primers were recommended by QBOL, and all primer sequences are shown in Table 1. The sequences for the commercially available *M13* (24 bp) sequencing primers were added to the 5' end of each primer (indicated by underlined nucleotides).

**Table 1:** Primers and PCR conditions used for DNA sequence amplifications in this study.

Gene	Primer name	Amplification primers (5'-3')	PCR Conditions	Amplicon size
16S rDNA	16s F	CGCCAGGGTTTTCCCAGTCACGACGCGTAGAGTTTGATCCTGGCTCAG	94°C 5 min; 94°C 1 min, 60°C 1 min, 72°C 1 min, 35 cycles; 72°C 10 min	1200 bp
	16s R	AGCGGATAACAATTTCACACAGGAGACGGGCGGTGTGTRCA		
*cpn60*	H1594	CGCCAGGGTTTTCCCAGTCACGACGACGTCGCCGGTGACGGCACCACCAC	94°C 5 min; 94°C 30 s, 57°C 30 s, 72°C 45 s, 35 cycles; 72°C 10 min	555 bp
	H1595	AGCGGATAACAATTTCACACAGGACGACGGTCGCCGAAGCCCGGGGCCTT		
*gyrB*	gyr-f	CGCCAGGGTTTTCCCAGTCACGACAAGCAGGGCAAGAGCGAGCTGTA	94°C 2.5 min; 94°C 30 s, 50°C 45 s, 72°C 1 min, 35 cycles; 72°C 7 min	600 bp
	gyr-r	AGCGGATAACAATTTCACACAGGACAAGGTGCTGAAGATCTGGTC		
*avrBs2*	AvrBs2-F	CGCCAGGGTTTTCCCAGTCACGACGGACTAGTCCTGCCGGTGTTGATGCACGA	94°C 2 min; 94°C 1 min, 60°C 1 min, 72°C 1 min, 35 cycles; 72°C 7 min	780 bp
	AvrBs2-R	AGCGGATAACAATTTCACACAGGACCGCTCGAGCGGTGATCGGTCAACAGGCTTTC		

PCR amplification of the four candidate barcodes was performed in 50 μL reaction mixtures containing 25 μL of 2× PCR Master Mix (Biomed Biotechnology, Beijing, China), 19 μL of ddH_2_O, 2 μL of each primer (10 μM), and 2 μL of template DNA. The amplification conditions for each region are provided in Table 1. PCR products were examined by 1.5% agarose gel electrophoresis. Purification and bidirectional sequencing were completed by Biomed Biotechnology, using the sequencing primers M13-47 and M13-48.

### Data analysis

Tree-based methods (Neighbor-joining; NJ) and distance-based methods (the ‘best close match’ and barcoding gap analysis) were applied to test the efficacy of the four DNA barcode candidates for the identification of quarantine *Xanthomonas* species/pathovars. Sequences were assembled and edited using DNAMAN version 7.0 software (Lynnon Corporation, Quebec, Canada). Multiple sequence alignments were performed using the ClustalW tool from MEGA 6.0 [17], with the default parameters (gap opening penalty of 15 and gap extension penalty of 6.66).

#### Tree-based methods

Species/pathovar discrimination was evaluated through tree-based analysis of each gene. NJ trees, which were recommended as the standard barcoding method [1], were adopted and constructed using MEGA 6.0 software, based on the Kimura 2-parameter (K2P) model. Branch support was evaluated with 1,000 bootstrap replicates.

#### Distance-based methods

Barcoding gap and ‘best close match’ analyses were conducted for each respective gene. Pairwise distances were calculated separately to determine the intra- and inter-taxon variation using Spider [18,19], Brown *et al*.’s DNA barcode analysis package for R, with the uncorrected K2P method. The presence of ‘barcoding gap’ between intra- and inter-taxon distances was evaluated using frequency histograms based on the uncorrected K2P distance. The distance-based criteria for ‘best close match’, which was proposed by Meier *et al*. (2006) [20], were also evaluated using Spider to estimate the identification success rate. The threshold similarity value was calculated separately for each matrix (all genes separately), below which 95% of all intra-taxon distances are found. In such tests, results can be classified as follows: if all matches of the query sequence belong to the same species/pathovar, the barcode assignment is recorded as a ‘correct’ identification; if a query sequence does not encounter a sequence of the same species/pathovar within the threshold, the test is recorded as an ‘incorrect’ identification; if the matches of the query sequence were equally good, but correspond to a mixture of species/pathovars (including the correct one) within the threshold, it is recorded as ‘ambiguous’; and no match within the threshold is reported as ‘no identification’.

To evaluate the DNA barcode candidates at the pathovar-level, the seven species included in our study that have been defined only to species-level were treated as equivalent to the other 43 pathovars. In other words, the 50 *Xanthomonas* species/pathovars were treated as 50 individual taxons. Thus, comparisons made within the same pathovar or species (only those seven that were not defined to the pathovar level) were considered as “intra-”, whereas comparisons made between the 50 individual taxons [e.g. between different pathovars (whether or not they are of the same species) or different species (only those seven that were not defined to the pathovar level), or between pathovars and those seven species] were considered as “inter-”.

## Results

### Amplification, sequencing success, and marker features

Each of the 327 strains was subjected to PCR analysis for the four DNA barcode candidates (16S rRNA gene, *cpn60*, *gyrB*, and *avrBs2*), respectively. The sequence information is provided in Table 2. The amplification reactions were performed with high success (>90%) for all four candidate genes. Specifically, *cpn60* exhibited the highest success rate (amplified from 100% of the strains tested), while *gyrB* (98%) and the 16S rRNA gene (97%) exhibited intermediate efficiency. In contrast, *avrBs2* showed the lowest efficiency, with a 93% success rate. High-quality sequences were obtained from all of the amplified DNA samples, and the four DNA regions chosen were effectively amplified and sequenced from the majority of the species/pathovars tested. Additionally, we obtained the 16S rRNA, *cpn60*, *gyrB*, and *avrBs2* gene sequences for 17 species/pathovars from GenBank (accession numbers shown in S2 Table), thereby generating a database of 1,380 sequences; however, it was not possible to obtain sequences for all of the DNA regions for all strains. All sequence files are available at the DNA Barcode Appraisal System on Chinese Quarantine Pests: http://www.qbol.org.cn/. Users can find or retrieve the data by searching for a species name in the integrated query link after registration for the service. Moreover, we also uploaded all the sequences to Figshare (https://figshare.com/s/31f44679024e443bc45f).

**Table 2:** Sample sizes, success rates of amplification and sequencing, and sequence characteristics of the four DNA regions in the *Xanthomonas* species assessed in this study.

Gene	Success rate of amplification (%)	Success rate of sequencing (%)	Alignment Length (bp)	No. of conserved sites	No. of variable sites	No. of parsimony informative sites
16S rRNA	97	100	1169	1120	49	39
*cpn60*	100	100	555	423	132	127
*gyrB*	98	100	623	357	266	260
*avrBs2*	93	100	797	448	349	311

For the quarantine *Xanthomonas*, *cpn60* was successfully amplified and sequenced from all 14 quarantine species/pathovars, whereas the 16S rRNA and *gyrB* genes were amplified from only 12 species/pathovars (failing for *X*. *albilineans* and *X*. *populi*) and the *avrBs2* gene from only eight species/pathovars (failing for *X*. *albilineans*, *X*. *axonopodis* pv. *manihotis*, *X*. *campestris* pv. *musacearum*, *X*. *fragariae*, X. *hyacinthi*, and *X*. *populi*).

For analysis convenience, the sequences for each candidate gene were trimmed to a uniform length that provided 100% coverage for each strain. The lengths of the aligned DNA fragments of the 16S rRNA, *cpn60*, *gyrB*, and *avrBs2* genes were 1,169, 555, 623, and 797 bp, respectively. Of the four regions amplified and sequenced, the 16S rRNA gene was the most highly conserved (1120/1169 nucleotides), based on both sequence length and the number of conserved sites. Meanwhile, the target region of *avrBs2* provided the largest number of variable sites (349) and parsimony-informative sites (311), followed by *gyrB* (266 and 260, respectively), *cpn60* (132 and 127, respectively), and the 16S rRNA gene (49 and 39, respectively).

### Phylogenetic tree-based discrimination of *Xanthomonas* at the species and pathovar level

**Fig 1:**
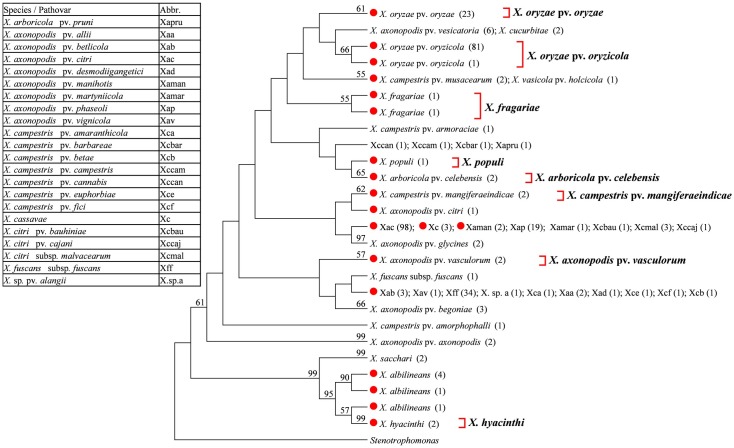
Neighbor-joining tree based on 16S rRNA gene. Bootstrap values (>50%) are shown above the branches. Identical sequences are represented only once. Numbers following taxon names indicate the number of isolates. Red dots indicate quarantine *Xanthomonas* species/pathovars. Species/pathovars that were successfully identified are shown on the right.

**Fig 2:**
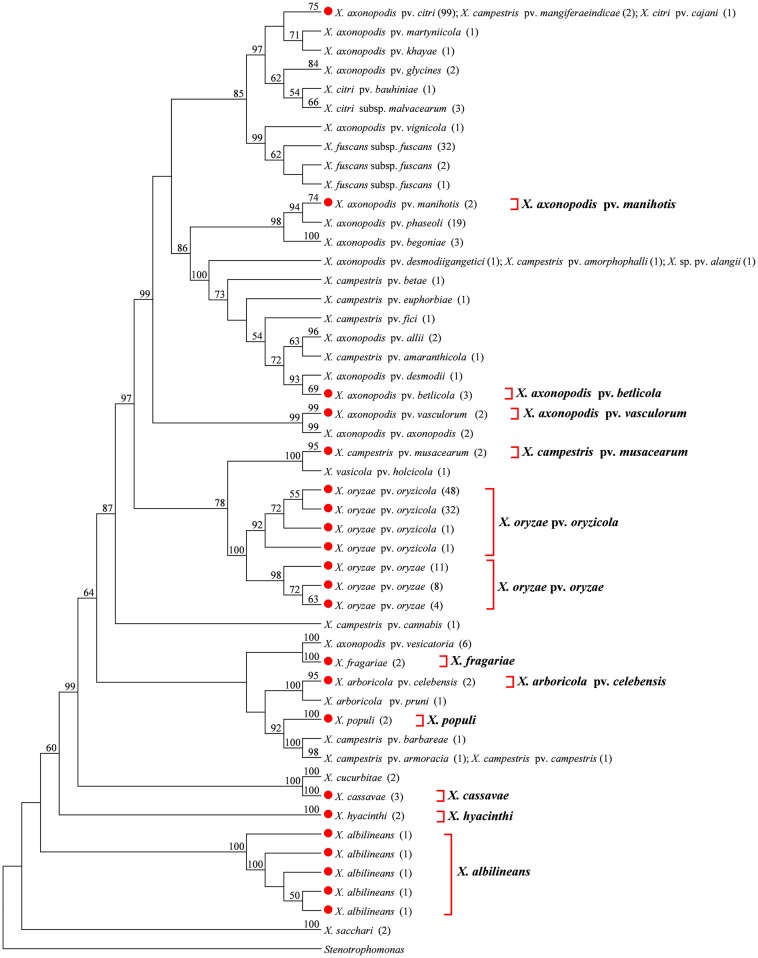
Neighbor-joining tree based on *gyrB*. Bootstrap values (>50%) are shown above the branches. Identical sequences are represented only once. Numbers following taxon names indicate the number of isolates. Red dots indicate quarantine *Xanthomonas* species/pathovars. Species/pathovars that were successfully identified are shown on the right.

**Fig 3:**
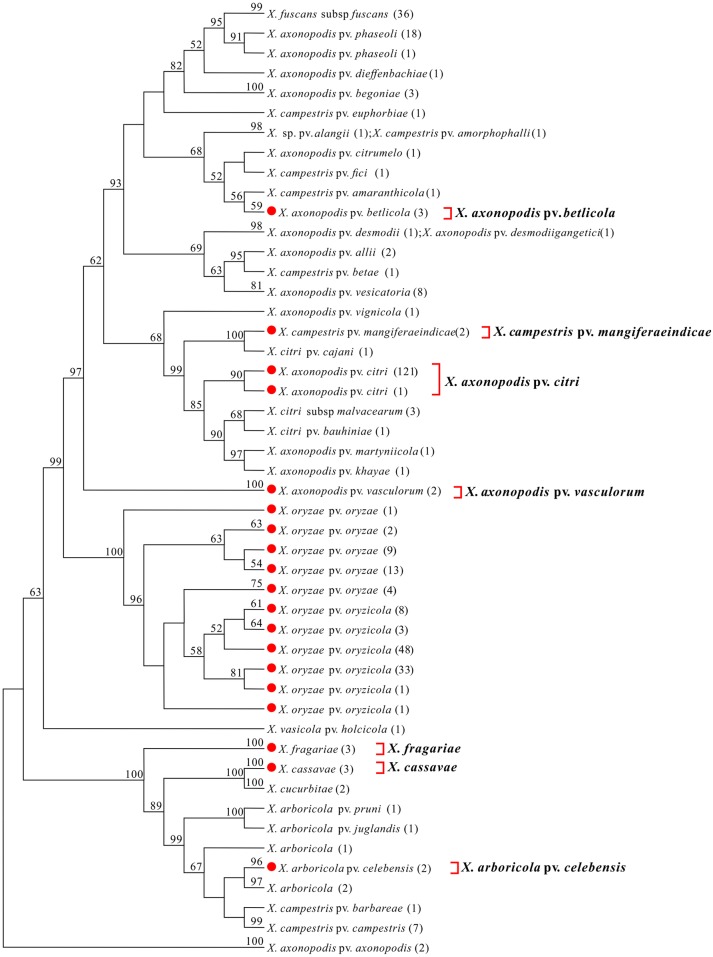
Neighbor-joining tree based on *avrBs2*. Bootstrap values (>50%) are shown above the branches. Identical sequences are represented only once. Numbers following taxon names indicate the number of isolates. Red dots indicate quarantine *Xanthomonas* species/pathovars. Species/pathovars that were successfully identified are shown on the right.

**Fig 4:**
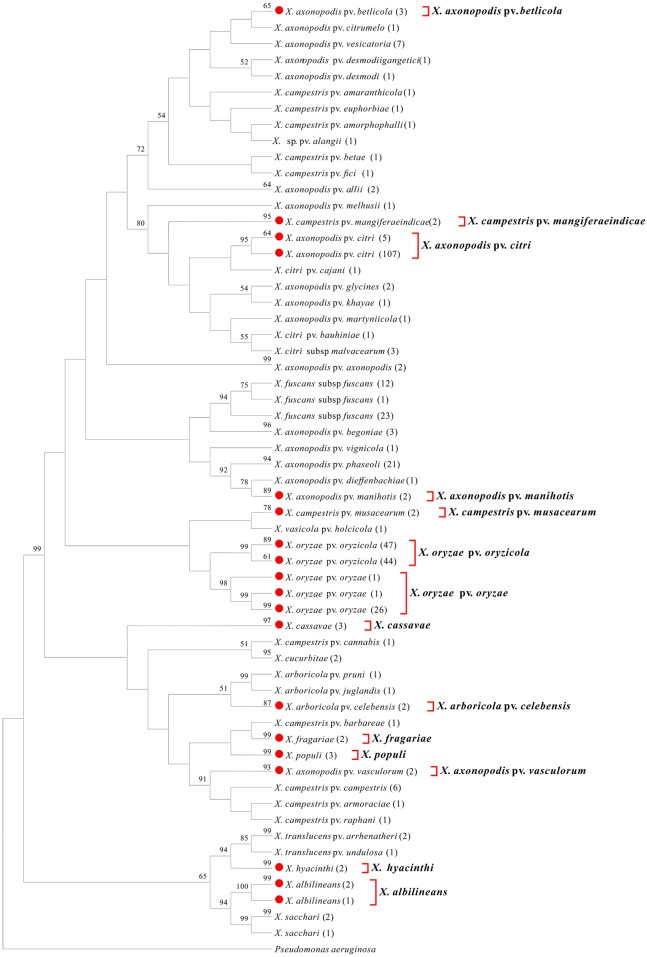
Neighbor-joining tree based on *cpn60*. Bootstrap values (>50%) are shown above the branches. Identical sequences are represented only once. Numbers following taxon names indicate the number of isolates. Red dots indicate quarantine *Xanthomonas* species/pathovars. Species/pathovars that were successfully identified are shown on the right.

We conducted a phylogenetic tree-based analysis to evaluate the effectiveness of the four DNA barcode candidate genes for species- and pathovar-level discrimination of *Xanthomonas* strains. Due to the large number of sequences used, the NJ tree is too large to be displayed in its entirety. We therefore used the “Compress Subtree” tool within the MEGA package to compress identical sequences within the same branch, respectively. As a result, such sequences are represented only once in Figs 1, 2, 3 and 4.

#### 16S rRNA

Amplification of 16S rRNA gene was unsuccessful for two quarantine *Xanthomonas* species, *X*. *albilineans* and *X*. *populi*. We therefore obtained an additional seven 16S rRNA gene sequences for these two species from GenBank. In total, 324 16S rRNA gene sequences, representing 39 *Xanthomonas* species/pathovars, were used in the present study. The 16S rRNA gene sequence from a species of *Stenotrophomonas* was chosen as the outgroup. NJ analysis successfully identified 8 of target quarantine species/pathovars, as these isolates were clustered together into a monophyletic group (Fig 1). However, sequences of *X*. *axonopodis* pv. *citri* (with the exception of one isolate), *X*. *axonopodis* pv. *manihotis*, and *X*. *cassavae* were identified as identical to five non-quarantine pathovars, while *X*. *campestris* pv. *musacearum* sequences were identical to *X*. *vasicola* pv. *holcicola*, and *X*. *axonopodis* pv. *betlicola* sequences were identical to 9 other non-quarantine pathovars. In these cases, quarantine and non-quarantine pathovars could not be distinguished. In addition, six *X*. *albilineans* isolates were assigned to distinct branches, meaning that they could not be clustered together into a monophyletic group.

#### gyrB

We failed to amplify *gyrB* from two quarantine *Xanthomonas* species, *X*. *albilineans and X*. *populi*. We therefore obtained an additional seven *gyrB* sequences for these two species from GenBank. Thus, a total of 326 *gyrB* sequences, representing 43 *Xanthomonas* species/pathovars, were used in the present study. The *gyrB* sequence of *Stenotrophomonas* was used as an out-group. While NJ analysis successfully identified 12 of the target quarantine species/pathovars, as isolates from these species/pathovars clustered together into a monophyletic group (Fig 2), *X*. *axonopodis* pv. *citri* and *X*. *campestris* pv. *mangiferaeindicae* represented complex pathovar clusters containing both quarantine and non-quarantine pathovars that could not be clearly distinguished.

#### avrBs2

Amplification of *avrBs2* was unsuccessful for six quarantine *Xanthomonas* species/pathovars: *X*. *albilineans*, *X*. *axonopodis pv*. *manihotis*, *X*. *campestris pv*. *musacearum*, *X*. *fragariae*, *X*. *hyacinthi*, and *X*. *populi*. Notably, only the *avrBs2* sequences of *X*. *fragariae* could be obtained from GenBank. However, we also obtained an additional 59 *avrBs2* sequences for 11 species/pathovars from GenBank. Thus, a total of 364 *avrBs2* sequences, representing 37 *Xanthomonas* species/pathovars (containing nine quarantine species/pathovars), were subjected to NJ analysis; the tree was rooted using *avrBs2* sequences from *X*. *axonopodis* pv. *axonopodis*. These analyses successfully identified seven target quarantine species/pathovars (Fig 3). However, the sequences from *X*. *oryzae pv*. *oryzae* and *X*. *oryzae pv*. *oryzicola* were clustered together into one group, which could not be clearly distinguished.

#### cpn60

Notably, we achieved successful amplification of *cpn60* from all 14 quarantine *Xanthomonas* species/pathovars. Moreover, an additional 43 *cpn60* sequences from 14 species/pathovars were obtained from GenBank. In total, 370 *cpn60* sequences, representing 50 *Xanthomonas* species/pathovars, were used in the present study. The *cpn60* sequence from *Pseudomonas aeruginosa* was chosen as an outgroup. NJ analysis successfully identified each of the 14 target quarantine species/pathovars, as the isolates of these species/pathovars clustered together into a highly supported monophyletic group (>90% bootstrap support, with the exception of *X*. *axonopodis* pv. *betlicola*, *X*. *axonopodis pv*. *manihotis*, and *X*. *campestris pv*. *musacearum*, which displayed bootstrap support values of 65%, 89%, and 78%, respectively), separating them from their closest relatives (Fig 4). Moreover, all *Xanthomonas* species/pathovars with multiple isolates were recovered as monophyletic. These data suggest that *cpn60* may be the most suitable target gene for use as a DNA barcode to distinguish quarantine species/pathovars of *Xanthomonas*.

### Distance-based discrimination of *Xanthomonas* at the species and pathovar level

**Fig 5:**
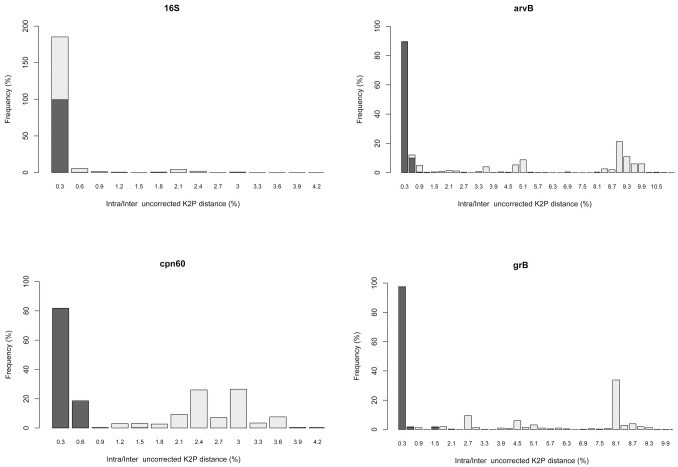
Histograms of the frequencies (y-axes) of pairwise intra-species/pathovar (dark gray bars) and inter-species/pathovar (light gray bars) divergences based on the uncorrected K2P distance (x-axes) for each candidate gene.

For a robust DNA barcode, it is essential that genetic variation between species be much larger than variation within species. Via calculation of uncorrected K2P distances, variations in intra- and inter-species/pathovar distances were observed among the four DNA barcode candidates. Meanwhile, the presence or absence of a barcoding gap for individual genes was represented by the frequency distribution of the genetic distance between intra-species/pathovar sequences and between inter-species/pathovar sequences (Fig 5). Among the four candidates, *cpn60* presented the best barcode gap performance, with 99.73% of pairwise intra-species/pathovar K2P distances lower than 0.006, and 99.25% of pairwise inter-species/pathovar K2P distances greater than 0.006. In addition, *avrBs2* performed quite well, providing the highest values for inter-species/pathovar genetic distances, with a range varying from zero to a maximum of 0.326. Notably, however, there was still a relatively high frequency of low distances at the inter-species/pathovar level, with only 97.57% of pairwise inter-species/pathovar K2P distances greater than 0.006, and 99.31% of pairwise intra-species/pathovar K2P distances lower than 0.006. Conversely, 16S rRNA gene showed the worst performance, with a total overlap of intra- and inter-species/pathovar variation, and the *gyrB* also performed unsatisfactorily.

The results of the ‘best close match’ test for each single gene revealed *avrBs2* and *cpn60* as the best at species/pathovars identification (94.94% and 92.96%, respectively; Table 3). In contrast, *gyrB* and the 16S rRNA gene exhibited poor rates of correct identification (62.77% and 39.63%, respectively) and larger proportions of ambiguous identifications (31.08% and 54.49%, respectively). Incorrect identification and no identification rates were relatively low (< 6%) for all of the genes tested. Our data contained a number of singleton species/pathovars, which likely led to a lower identification success rate for the candidate genes. To evaluate how the data might behave once fully sampled, we also performed a ‘best close match’ test for each single gene after removing these singletons. The results revealed a higher correct identification rate and a lower incorrect identification rate for all of the individual genes. In particular, the rate of correct identification for *cpn60* and *avrBs2* were both >99%. Additionally, we also tested the effectiveness of selected gene combinations (*cpn60* + *gyrB*, *cpn60* + *gyrB* + *avrBs2*, and *cpn60* + *gyrB* + *avrBs2* + 16S rRNA) for species identification using the distance-based methods. While each of the three combinations performed satisfactorily in the barcoding gap analysis and the ‘best close match’ test (data not shown), neither provided a decided advantage over the *cpn60* gene gene alone.

**Table 3:** Identification success based on “best close match” method.

	best close match (include singleton)				best close match (exclude singleton)			
Gene	Correct	Incorrect	Ambiguous	No id	Correct	Incorrect	Ambiguous	No id
16S rRNA	39.63%	5.58%	54.49%	0.31%	41.83%	0.33%	57.52%	0.33%
*cpn60*	92.96%	5.97%	0.00%	1.09%	99.14%	0.00%	0.00%	0.87%
*gyrB*	62.77%	4.31%	31.08%	1.85%	66.45%	0.00%	32.9%	0.66%
*avrBs2*	94.94%	3.86%	0.00%	1.66%	99.42%	0.00%	0.00%	0.58%

## Discussion

Development of a method for accurate DNA barcoding of *Xanthomonas* is essential, as it would provide a rapid and accurate means for discrimination of *Xanthomonas* strains and would assist in screening for quarantine pathogens. In general, there were notable differences in the abilities of the different candidate gene sequences to distinguish between different groups of bacteria. For *Xanthomonas*, QBOL recommends using the 16S rRNA, *gyrB*, and *avrBs2* genes as core barcode genes for ensuring accurate identification. Of these, the 16S rRNA gene is the most commonly used for bacterial identification. Molecular phylogenetics based on this ribosomal RNA gene has played a vital role in the classification of the genus *Xanthomonas*; however, the results of our study demonstrate that it is not suitable for identification below the species level due to its low resolution. Specifically, while our data show that this gene can be used to successfully distinguish *Xanthomonas* spp. from the closely related genus *Stenotrophomonas*, it failed to provide sufficient information for species-level identification. Moreover, this gene also demonstrated poor performance in the barcoding gap analysis and the ‘best close match’ test.

In previous studies, Parkinson *et al*. (2008 & 2009) [10,16] evaluated the applicability of *gyrB* sequences for species and intra-species identification in *Xanthomonas*, noting that the *gyrB* sequences used were sufficiently distinct to discriminate between the majority of established *Xanthomonas* species, thereby offering greater resolution than the 16S rRNA gene [21]. To further evaluate this locus for quarantine species/pathovar identification, we tested a large number of quarantine species/pathovar strains via the NJ method. Our results demonstrate that, based on a partial sequence of the *gyrB* gene (approximately 700 bp), it is possible to identify most of the quarantine species/pathovars (*X*. *albilineans*, *X*. *arboricola* pv. *celebensis*, *X*. *axonopodis* pv. *betlicola*, *X*. *axonopodis* pv. *manihotis*, *X*. *axonopodis* pv. *vasculorum*, *X*. *campestris* pv. *musacearum*, *X*. *cassavae*, *X*. *fragariae*, *X*. *hyacinthi*, *X*. *oryzae* pv. *oryzae*, *X*. *oryzae* pv. *oryzicola*, and *X*. *populi*). However, this gene was incapable of identifying *Xanthomonas axonopodis* pv. *citri* and *X*. *campestris* pv. *mangiferaeindicae*, which were grouped within complex pathovar clusters containing both quarantine and non-quarantine pathovars. Additional genes would therefore be necessary to identify the quarantine pathovars within the complex clusters. Moreover, *gyrB* performed markedly worse than *cpn60* and *avrBs2* in the barcoding gap analysis and ‘‘best close match” test. Together, these data demonstrate that *gyrB* does not live up to our expectations for pathovar-level identification of *Xanthomonas* strains.

The T3E gene *avrBs2* is broadly distributed among *Xanthomonas* strains. Hajri *et al*. (2009) [13] detected a correlation between the composition of T3E repertoires and pathovars of *X*. *axonopodis*. We therefore further evaluated the applicability of this locus for use in quarantine species/pathovars identification. Despite multiple attempts, we were unable to amplify the *avrBs2*sequence from five quarantine species/pathovars (*X*. *albilineans*, *X*. *axonopodis pv*. *manihotis*, *X*. *campestris pv*. *musacearum*, *X*. *hyacinthi*, and *X*. *populi*), resulting in a markedly lower reaction efficiency for quarantine species/pathovars than the other loci tested. This effect could be due to the high sequence variability observed for this gene in our study. Notably, however, *avrBs2* still performed better than the 16S rRNA and *gyrB* genes for species- and pathovar-level identification of *Xanthomonas*. Specifically, we successfully identified seven of the nine quarantine species/pathovars tested (*X*. *arboricola* pv. *celebensis*, *X*. *axonopodis* pv. *betlicola*, *X*. *axonopodis* pv. *citri*, *X*. *axonopodis* pv. *vasculorum*, *X*. *campestris* pv. *mangiferaeindicae*, *X*. *cassavae*, and *X*. *fragariae*) via phylogenetic analysis using a partial sequence of the *avrBs2* gene (approximately 800 bp). Moreover, *avrBs2* performed satisfactorily in the barcoding gap analysis and the ‘best close match’ test, exhibiting the highest inter-species/pathovar genetic distance values, a small overlap of intra- and inter-species/pathovar variation, and the highest correct identification rate. While the efficacy of *avrBs2* for species and pathovar identification indicates that the this gene might comprise an ideal candidate for DNA barcoding, the lack of a set of efficient universal primers for amplification *avrBs2* constitutes a major limitation for the application of this locus for such testing. Therefore, further research should be conducted to develop efficient universal primers for the *avrBs2* gene.

*cpn60*, which encodes the 60 kDa chaperonin protein, has been established as a target for the detection, identification, and classification of many microorganisms [22,23,24,25,26,27,28,29,30]. Previously, Links *et al*. (2010) [14] performed a barcode analysis of *cpn60* and several regions of the 16S rRNA gene, and found that the *cpn60* universal target exhibited a much larger barcode gap than 16S rRNA gene, suggesting *cpn60* as a preferred barcode gene for bacteria. In this study, we evaluated the usefulness of this locus for the identification of quarantine *Xanthomonas* species/pathovars. The efficiency of PCR amplification and sequencing is an important index for evaluating a candidate DNA barcode. In this regard, the *cpn60* gene exhibited the highest success rate of amplification (100%) and sequencing (100%) of the four genes tested in our study. Moreover, NJ tree analysis of a region of this gene (approximately 555 bp) enabled successful identification of all 14 target quarantine species/pathovars, as isolates were clustered together into a highly supported monophyletic group, separating them from their closest relatives. Furthermore, along with the quarantine species/pathovars, another 12 non-quarantine species/pathovars, represented by multiple isolates, were recovered as monophyletic. Meanwhile, the remaining 24 species/pathovars, represented by only one isolate each, were all located in independent branch. Moreover, the *cpn60* gene provided the most efficient identification of *Xanthomonas* species/pathovars, exhibiting the best barcode gap performance and a high identification rate (99.14% with the singletons removed) when using all of the sequences obtained (n = 370). Thus, it can be inferred that the *cpn60* gene might be suitable for use as a DNA barcode for all species/pathovars of the genus *Xanthomonas*. However, further research will be required to verify this assertion. Besides, *cpn60* alone yielded similar results to combinations of candidate barcode genes, indicating that this locus is sufficient for rapid and accurate discrimination of *Xanthomonas* strains. Coupled with the low amplification success rate for the other three genes, we feel that it is not necessary to use combinations of genes as a DNA barcode for quarantine *Xanthomonas* strains.

In summary, an ideal DNA barcode should be universal, reliable, and show good discriminatory power. Based on the amplification and sequencing efficiency, the intra- and inter-species/pathovar divergence patterns, the ‘best close match’ test results, and the ability to recover species/pathovar-specific clusters in phylogenetic trees observed in this study, we recommend *cpn60* for use in barcode identification of the quarantine pathogen *Xanthomonas*.

## Supporting Information

S1 Table Strains of *Xanthomonas* used in this study. (PDF)

S2 Table Additional *Xanthomonas* sequences obtained from GenBank. (PDF)
