# Supplementary material for: DNA Barcoding for Efficient Species- and Pathovar-Level Identification of the Quarantine Plant Pathogen Xanthomonas
Source: PLoS One. 2016 Nov 18;11(11):e0165995. doi: 10.1371/journal.pone.0165995 (PMC5115671; doi:10.1371/journal.pone.0165995)
Supplement: S1 Table — (PDF) [file pone.0165995.s001.pdf]

S1 Table. Strains of *Xanthomonas* used in this study.

No.	Species/Pathovar	Strain	Source
1	*Xanthomonas albilineans*	DSM 3583	DSMZ
2	*Xanthomonas albilineans*	ICMP 196	ICMP
3	*Xanthomonas arboricola* pv. *pruni*	ATCC 15924	ATCC
4	*Xanthomonas arboricola* pv. *celebensis*	ATCC 19045	ATCC
5	*Xanthomonas arboricola* pv. *celebensis*	DSM 50853	DSMZ
6	*Xanthomonas axonopodis* pv. *allii*	LMG 578	LMG
7	*Xanthomonas axonopodis* pv. *allii*	LMG 576	LMG
8	*Xanthomonas axonopodis* pv. *axonopodis*	DSM 3585	DSMZ
9	*Xanthomonas axonopodis* pv. *axonopodis*	LMG 539	LMG
10	*Xanthomonas axonopodis* pv. *begoniae*	DSM 50850	DSMZ
11	*Xanthomonas axonopodis* pv. *begoniae*	LMG 550	LMG
12	*Xanthomonas axonopodis* pv. *begoniae*	LMG 553	LMG
13	*Xanthomonas axonopodis* pv. *betlicola*	ATCC 11677	ATCC
14	*Xanthomonas axonopodis* pv. *betlicola*	LMG 555	LMG
15	*Xanthomonas axonopodis* pv. *betlicola*	ICMP 312	ICMP
16	*Xanthomonas axonopodis* pv. *citri*	1	CAIQ
17	*Xanthomonas axonopodis* pv. *citri*	2	CAIQ
18	*Xanthomonas axonopodis* pv. *citri*	3	CAIQ
19	*Xanthomonas axonopodis* pv. *citri*	4	CAIQ
20	*Xanthomonas axonopodis* pv. *citri*	5	CAIQ
21	*Xanthomonas axonopodis* pv. *citri*	6	CAIQ
22	*Xanthomonas axonopodis* pv. *citri*	7	CAIQ
23	*Xanthomonas axonopodis* pv. *citri*	8	CAIQ
24	*Xanthomonas axonopodis* pv. *citri*	9	CAIQ
25	*Xanthomonas axonopodis* pv. *citri*	11	CAIQ
26	*Xanthomonas axonopodis* pv. *citri*	12	CAIQ
27	*Xanthomonas axonopodis* pv. *citri*	FZ01	CAIQ
28	*Xanthomonas axonopodis* pv. *citri*	GJ1-Y-2	CAIQ
29	*Xanthomonas axonopodis* pv. *citri*	GJ2-Y-3	CAIQ
30	*Xanthomonas axonopodis* pv. *citri*	GJ3-G-1	CAIQ
31	*Xanthomonas axonopodis* pv. *citri*	X206-5	CAIQ
32	*Xanthomonas axonopodis* pv. *citri*	Xac-02	CAIQ
33	*Xanthomonas axonopodis* pv. *citri*	YG201	CAIQ
34	*Xanthomonas axonopodis* pv. *citri*	YG202	CAIQ
35	*Xanthomonas axonopodis* pv. *citri*	YG206	CAIQ
36	*Xanthomonas axonopodis* pv. *citri*	YG208	CAIQ
37	*Xanthomonas axonopodis* pv. *citri*	YG209	CAIQ
38	*Xanthomonas axonopodis* pv. *citri*	YG210	CAIQ
39	*Xanthomonas axonopodis* pv. *citri*	YG212	CAIQ
40	*Xanthomonas axonopodis* pv. *citri*	YG213	CAIQ

No.	Species/Pathovar	Strain	Source
41	*Xanthomonas axonopodis* pv. *citri*	YG214	CAIQ
42	*Xanthomonas axonopodis* pv. *citri*	YG215	CAIQ
43	*Xanthomonas axonopodis* pv. *citri*	YG220	CAIQ
44	*Xanthomonas axonopodis* pv. *citri*	YG221	CAIQ
45	*Xanthomonas axonopodis* pv. *citri*	YG222	CAIQ
46	*Xanthomonas axonopodis* pv. *citri*	YG223	CAIQ
47	*Xanthomonas axonopodis* pv. *citri*	YG226	CAIQ
48	*Xanthomonas axonopodis* pv. *citri*	YG230	CAIQ
49	*Xanthomonas axonopodis* pv. *citri*	YG231	CAIQ
50	*Xanthomonas axonopodis* pv. *citri*	YG232	CAIQ
51	*Xanthomonas axonopodis* pv. *citri*	YG233	CAIQ
52	*Xanthomonas axonopodis* pv. *citri*	YG234	CAIQ
53	*Xanthomonas axonopodis* pv. *citri*	YG235	CAIQ
54	*Xanthomonas axonopodis* pv. *citri*	YG236	CAIQ
55	*Xanthomonas axonopodis* pv. *citri*	YG237	CAIQ
56	*Xanthomonas axonopodis* pv. *citri*	YG238	CAIQ
57	*Xanthomonas axonopodis* pv. *citri*	YG239	CAIQ
58	*Xanthomonas axonopodis* pv. *citri*	YG240	CAIQ
59	*Xanthomonas axonopodis* pv. *citri*	YG242	CAIQ
60	*Xanthomonas axonopodis* pv. *citri*	YG243	CAIQ
61	*Xanthomonas axonopodis* pv. *citri*	YG245	CAIQ
62	*Xanthomonas axonopodis* pv. *citri*	YG246	CAIQ
63	*Xanthomonas axonopodis* pv. *citri*	YG247	CAIQ
64	*Xanthomonas axonopodis* pv. *citri*	YG248	CAIQ
65	*Xanthomonas axonopodis* pv. *citri*	YG250	CAIQ
66	*Xanthomonas axonopodis* pv. *citri*	YG251	CAIQ
67	*Xanthomonas axonopodis* pv. *citri*	YG252	CAIQ
68	*Xanthomonas axonopodis* pv. *citri*	YG253	CAIQ
69	*Xanthomonas axonopodis* pv. *citri*	YG254	CAIQ
70	*Xanthomonas axonopodis* pv. *citri*	YG255	CAIQ
71	*Xanthomonas axonopodis* pv. *citri*	YG256	CAIQ
72	*Xanthomonas axonopodis* pv. *citri*	YG257	CAIQ
73	*Xanthomonas axonopodis* pv. *citri*	YG258	CAIQ
74	*Xanthomonas axonopodis* pv. *citri*	YG259	CAIQ
75	*Xanthomonas axonopodis* pv. *citri*	YG260	CAIQ
76	*Xanthomonas axonopodis* pv. *citri*	YG261	CAIQ
77	*Xanthomonas axonopodis* pv. *citri*	YG262	CAIQ
78	*Xanthomonas axonopodis* pv. *citri*	YG264	CAIQ
79	*Xanthomonas axonopodis* pv. *citri*	YG265	CAIQ
80	*Xanthomonas axonopodis* pv. *citri*	YG266	CAIQ
81	*Xanthomonas axonopodis* pv. *citri*	YG267	CAIQ
82	*Xanthomonas axonopodis* pv. *citri*	YG270	CAIQ

No.	Species/Pathovar	Strain	Source
83	*Xanthomonas axonopodis* pv. *citri*	YG271	CAIQ
84	*Xanthomonas axonopodis* pv. *citri*	YG272	CAIQ
85	*Xanthomonas axonopodis* pv. *citri*	YG274	CAIQ
86	*Xanthomonas axonopodis* pv. *citri*	YG275	CAIQ
87	*Xanthomonas axonopodis* pv. *citri*	YG276	CAIQ
88	*Xanthomonas axonopodis* pv. *citri*	YG277	CAIQ
89	*Xanthomonas axonopodis* pv. *citri*	YG278	CAIQ
90	*Xanthomonas axonopodis* pv. *citri*	YG279	CAIQ
91	*Xanthomonas axonopodis* pv. *citri*	YG280	CAIQ
92	*Xanthomonas axonopodis* pv. *citri*	YG281	CAIQ
93	*Xanthomonas axonopodis* pv. *citri*	YG282	CAIQ
94	*Xanthomonas axonopodis* pv. *citri*	YG283	CAIQ
95	*Xanthomonas axonopodis* pv. *citri*	YG284	CAIQ
96	*Xanthomonas axonopodis* pv. *citri*	YG285	CAIQ
97	*Xanthomonas axonopodis* pv. *citri*	YG286	CAIQ
98	*Xanthomonas axonopodis* pv. *citri*	YG287	CAIQ
99	*Xanthomonas axonopodis* pv. *citri*	YG288	CAIQ
100	*Xanthomonas axonopodis* pv. *citri*	YG289	CAIQ
101	*Xanthomonas axonopodis* pv. *citri*	YG290	CAIQ
102	*Xanthomonas axonopodis* pv. *citri*	YG292	CAIQ
103	*Xanthomonas axonopodis* pv. *citri*	YG293	CAIQ
104	*Xanthomonas axonopodis* pv. *citri*	YG294	CAIQ
105	*Xanthomonas axonopodis* pv. *citri*	YG295	CAIQ
106	*Xanthomonas axonopodis* pv. *citri*	YG296	CAIQ
107	*Xanthomonas axonopodis* pv. *citri*	YG297	CAIQ
108	*Xanthomonas axonopodis* pv. *citri*	YG298	CAIQ
109	*Xanthomonas axonopodis* pv. *citri*	YG299	CAIQ
110	*Xanthomonas axonopodis* pv. *citri*	YG300	CAIQ
111	*Xanthomonas axonopodis* pv. *citri*	YG301	CAIQ
112	*Xanthomonas axonopodis* pv. *citri*	YG302	CAIQ
113	*Xanthomonas axonopodis* pv. *citri*	YG303	CAIQ
114	*Xanthomonas axonopodis* pv. *citri*	YG306	CAIQ
115	*Xanthomonas axonopodis* pv. *desmodii*	LMG 692	LMG
116	*Xanthomonas axonopodis* pv. *desmodiigangetici*	LMG 693	LMG
117	*Xanthomonas axonopodis* pv. *glycines*	ATCC 43911	ATCC
118	*Xanthomonas axonopodis* pv. *glycines*	ICMP 5732	ICMP
119	*Xanthomonas axonopodis* pv. *khayae*	NCPPB 536	NCPPB
120	*Xanthomonas axonopodis* pv. *manihotis*	ATCC 23380	ATCC
121	*Xanthomonas axonopodis* pv. *manihotis*	ICMP 5741	ICMP
122	*Xanthomonas axonopodis* pv. *martyniicola*	NCPPB 1148	NCPPB
123	*Xanthomonas axonopodis* pv. *melhusii*	NCPPB 479	NCPPB
124	*Xanthomonas axonopodis* pv. *phaseoli*	ATCC 49119	ATCC

No.	Species/Pathovar	Strain	Source
125	*Xanthomonas axonopodis* pv. *phaseoli*	JC2	CAIQ
126	*Xanthomonas axonopodis* pv. *phaseoli*	JC6	CAIQ
127	*Xanthomonas axonopodis* pv. *phaseoli*	SX1-1	CAIQ
128	*Xanthomonas axonopodis* pv. *phaseoli*	SX1-2	CAIQ
129	*Xanthomonas axonopodis* pv. *phaseoli*	SX2-3	CAIQ
130	*Xanthomonas axonopodis* pv. *phaseoli*	YFS13-1	CAIQ
131	*Xanthomonas axonopodis* pv. *phaseoli*	YFS13-2	CAIQ
132	*Xanthomonas axonopodis* pv. *phaseoli*	YFS13-4	CAIQ
133	*Xanthomonas axonopodis* pv. *phaseoli*	YFS13-5	CAIQ
134	*Xanthomonas axonopodis* pv. *phaseoli*	YFS15-1	CAIQ
135	*Xanthomonas axonopodis* pv. *phaseoli*	YFS15-2	CAIQ
136	*Xanthomonas axonopodis* pv. *phaseoli*	YFS15-3	CAIQ
137	*Xanthomonas axonopodis* pv. *phaseoli*	YFS15-4	CAIQ
138	*Xanthomonas axonopodis* pv. *phaseoli*	YFS15-5	CAIQ
139	*Xanthomonas axonopodis* pv. *phaseoli*	YJX1-4	CAIQ
140	*Xanthomonas axonopodis* pv. *phaseoli*	YJX1-5	CAIQ
141	*Xanthomonas axonopodis* pv. *phaseoli*	YJX1-7	CAIQ
142	*Xanthomonas axonopodis* pv. *phaseoli*	ICMP 5834	ICMP
143	*Xanthomonas axonopodis* pv. *vasculorum*	ATCC 13901	ATCC
144	*Xanthomonas axonopodis* pv. *vasculorum*	ICMP 5757	ICMP
145	*Xanthomonas axonopodis* pv. *vesicatoria*	NCPPB 1438	NCPPB
146	*Xanthomonas axonopodis* pv. *vesicatoria*	NCPPB 2572	NCPPB
147	*Xanthomonas axonopodis* pv. *vesicatoria*	NCPPB 2574	NCPPB
148	*Xanthomonas axonopodis* pv. *vesicatoria*	NCPPB 2594	NCPPB
149	*Xanthomonas axonopodis* pv. *vesicatoria*	NCPPB 936	NCPPB
150	*Xanthomonas axonopodis* pv. *vesicatoria*	NCPPB 941	NCPPB
151	*Xanthomonas axonopodis* pv. *vignicola*	ATCC 11648	ATCC
152	*Xanthomonas campestris* pv. *amaranthicola*	LMG 498	LMG
153	*Xanthomonas campestris* pv. *amorphophalli*	LMG 499	LMG
154	*Xanthomonas campestris* pv. *armoraciae*	LMG 535	LMG
155	*Xanthomonas campestris* pv. *barbareae*	LMG 547	LMG
156	*Xanthomonas campestris* pv. *betae*	NCPPB 2592	NCPPB
157	*Xanthomonas campestris* pv. *campestris*	NCPPB 528	NCPPB
158	*Xanthomonas campestris* pv. *cannabis*	NCPPB 2876	NCPPB
159	*Xanthomonas campestris* pv. *euphorbiae*	NCPPB 1828	NCPPB
160	*Xanthomonas campestris* pv. *fici*	NCPPB 2372	NCPPB
161	*Xanthomonas campestris* pv. *mangiferaeindicae*	ATCC 11637	ATCC
162	*Xanthomonas campestris* pv. *mangiferaeindicae*	ICMP 5740	ICMP
163	*Xanthomonas campestris* pv. *musacearum*	ATCC 49084	ATCC
164	*Xanthomonas campestris* pv. *musacearum*	ICMP 2870	ICMP
165	*Xanthomonas cassavae*	ICMP 204	ICMP
166	*Xanthomonas cassavae*	LMG 671.1	LMG

No.	Species/Pathovar	Strain	Source
167	*Xanthomonas cassavae*	LMG 673	LMG
168	*Xanthomonas citri* pv. *bauhiniae*	LMG 548	LMG
169	*Xanthomonas citri* pv. *cajani*	LMG 558	LMG
170	*Xanthomonas citri* subsp. *malvacearum*	ATCC 12131	ATCC
171	*Xanthomonas citri* subsp. *malvacearum*	DSM 1220	DSMZ
172	*Xanthomonas citri* subsp. *malvacearum*	DSM 3849	DSMZ
173	*Xanthomonas cucurbitae*	NCPPB 2597	NCPPB
174	*Xanthomonas cucurbitae*	NCPPB 3168	NCPPB
175	*Xanthomonas fragariae*	ATCC 29076	ATCC
176	*Xanthomonas fragariae*	NCPPB1469	NCPPB
177	*Xanthomonas fuscans* subsp. *fuscans*	BH1-1	CAIQ
178	*Xanthomonas fuscans* subsp. *fuscans*	CB1	CAIQ
179	*Xanthomonas fuscans* subsp. *fuscans*	CB2	CAIQ
180	*Xanthomonas fuscans* subsp. *fuscans*	CB3	CAIQ
181	*Xanthomonas fuscans* subsp. *fuscans*	CB4	CAIQ
182	*Xanthomonas fuscans* subsp. *fuscans*	G2-H2	CAIQ
183	*Xanthomonas fuscans* subsp. *fuscans*	G2-H5	CAIQ
184	*Xanthomonas fuscans* subsp. *fuscans*	G2-H6	CAIQ
185	*Xanthomonas fuscans* subsp. *fuscans*	G2-R10	CAIQ
186	*Xanthomonas fuscans* subsp. *fuscans*	G2-R11	CAIQ
187	*Xanthomonas fuscans* subsp. *fuscans*	G2-R12	CAIQ
188	*Xanthomonas fuscans* subsp. *fuscans*	G2-R13	CAIQ
189	*Xanthomonas fuscans* subsp. *fuscans*	G2-R6	CAIQ
190	*Xanthomonas fuscans* subsp. *fuscans*	G2-R8	CAIQ
191	*Xanthomonas fuscans* subsp. *fuscans*	G2-X1	CAIQ
192	*Xanthomonas fuscans* subsp. *fuscans*	G2-X2	CAIQ
193	*Xanthomonas fuscans* subsp. *fuscans*	G2-X3	CAIQ
194	*Xanthomonas fuscans* subsp. *fuscans*	G2-X4	CAIQ
195	*Xanthomonas fuscans* subsp. *fuscans*	G2-X5	CAIQ
196	*Xanthomonas fuscans* subsp. *fuscans*	G2-X8	CAIQ
197	*Xanthomonas fuscans* subsp. *fuscans*	G2-Y11	CAIQ
198	*Xanthomonas fuscans* subsp. *fuscans*	G2-Y5	CAIQ
199	*Xanthomonas fuscans* subsp. *fuscans*	G2-Y6	CAIQ
200	*Xanthomonas fuscans* subsp. *fuscans*	G2-Y7	CAIQ
201	*Xanthomonas fuscans* subsp. *fuscans*	G2-Y9	CAIQ
202	*Xanthomonas fuscans* subsp. *fuscans*	wilt2-1	CAIQ
203	*Xanthomonas fuscans* subsp. *fuscans*	wilt2-2	CAIQ
204	*Xanthomonas fuscans* subsp. *fuscans*	wilt2-3	CAIQ
205	*Xanthomonas fuscans* subsp. *fuscans*	wilt2'-4	CAIQ
206	*Xanthomonas fuscans* subsp. *fuscans*	ZB1-1	CAIQ
207	*Xanthomonas fuscans* subsp. *fuscans*	ZB1-2	CAIQ
208	*Xanthomonas fuscans* subsp. *fuscans*	ZB2-2	CAIQ

No.	Species/Pathovar	Strain	Source
209	*Xanthomonas fuscans* subsp. *fuscans*	ZB2-3	CAIQ
210	*Xanthomonas fuscans* subsp. *fuscans*	ZH4-1	CAIQ
211	*Xanthomonas fuscans* subsp. *fuscans*	ZH4-2	CAIQ
212	*Xanthomonas hyacinthi*	DSM 50855	DSMZ
213	*Xanthomonas hyacinthi*	ATCC 12612	ATCC
214	*Xanthomonas oryzae* pv. *oryzae*	13LL115	CAIQ
215	*Xanthomonas oryzae* pv. *oryzae*	13LL124	CAIQ
216	*Xanthomonas oryzae* pv. *oryzae*	13LL125	CAIQ
217	*Xanthomonas oryzae* pv. *oryzae*	14LL116	CAIQ
218	*Xanthomonas oryzae* pv. *oryzae*	14LL141	CAIQ
219	*Xanthomonas oryzae* pv. *oryzae*	14LL142	CAIQ
220	*Xanthomonas oryzae* pv. *oryzae*	14LL143	CAIQ
221	*Xanthomonas oryzae* pv. *oryzae*	14LL144	CAIQ
222	*Xanthomonas oryzae* pv. *oryzae*	14LL145	CAIQ
223	*Xanthomonas oryzae* pv. *oryzae*	14LL149	CAIQ
224	*Xanthomonas oryzae* pv. *oryzae*	14LL150	CAIQ
225	*Xanthomonas oryzae* pv. *oryzae*	14LL153	CAIQ
226	*Xanthomonas oryzae* pv. *oryzae*	14LL155	CAIQ
227	*Xanthomonas oryzae* pv. *oryzae*	14LL168	CAIQ
228	*Xanthomonas oryzae* pv. *oryzae*	14LL177	CAIQ
229	*Xanthomonas oryzae* pv. *oryzae*	14LL180	CAIQ
230	*Xanthomonas oryzae* pv. *oryzae*	14LL183	CAIQ
231	*Xanthomonas oryzae* pv. *oryzae*	14LL185	CAIQ
232	*Xanthomonas oryzae* pv. *oryzae*	GYC627	CAIQ
233	*Xanthomonas oryzae* pv. *oryzae*	GYC629	CAIQ
234	*Xanthomonas oryzae* pv. *oryzae*	RS50	CAIQ
235	*Xanthomonas oryzae* pv. *oryzae*	RS61	CAIQ
236	*Xanthomonas oryzae* pv. *oryzae*	PX009A	CAIQ
237	*Xanthomonas oryzae* pv. *oryzicola*	09YNB9-43	CAIQ
238	*Xanthomonas oryzae* pv. *oryzicola*	10CGY101	CAIQ
239	*Xanthomonas oryzae* pv. *oryzicola*	13LL105	CAIQ
240	*Xanthomonas oryzae* pv. *oryzicola*	13LL107	CAIQ
241	*Xanthomonas oryzae* pv. *oryzicola*	13LL121	CAIQ
242	*Xanthomonas oryzae* pv. *oryzicola*	AHB2-11	CAIQ
243	*Xanthomonas oryzae* pv. *oryzicola*	CGY103	CAIQ
244	*Xanthomonas oryzae* pv. *oryzicola*	CGY104	CAIQ
245	*Xanthomonas oryzae* pv. *oryzicola*	CGY105	CAIQ
246	*Xanthomonas oryzae* pv. *oryzicola*	CGY106	CAIQ
247	*Xanthomonas oryzae* pv. *oryzicola*	CGY107	CAIQ
248	*Xanthomonas oryzae* pv. *oryzicola*	CGY108	CAIQ
249	*Xanthomonas oryzae* pv. *oryzicola*	CGY109	CAIQ
250	*Xanthomonas oryzae* pv. *oryzicola*	CGY111	CAIQ

No.	Species/Pathovar	Strain	Source
251	*Xanthomonas oryzae* pv. *oryzicola*	CGY112	CAIQ
252	*Xanthomonas oryzae* pv. *oryzicola*	CGY113	CAIQ
253	*Xanthomonas oryzae* pv. *oryzicola*	CGY116	CAIQ
254	*Xanthomonas oryzae* pv. *oryzicola*	CGY123	CAIQ
255	*Xanthomonas oryzae* pv. *oryzicola*	CGY124	CAIQ
256	*Xanthomonas oryzae* pv. *oryzicola*	CGY125	CAIQ
257	*Xanthomonas oryzae* pv. *oryzicola*	CGY126	CAIQ
258	*Xanthomonas oryzae* pv. *oryzicola*	CGY127	CAIQ
259	*Xanthomonas oryzae* pv. *oryzicola*	CGY128	CAIQ
260	*Xanthomonas oryzae* pv. *oryzicola*	CGY129	CAIQ
261	*Xanthomonas oryzae* pv. *oryzicola*	CGY130	CAIQ
262	*Xanthomonas oryzae* pv. *oryzicola*	CGY131	CAIQ
263	*Xanthomonas oryzae* pv. *oryzicola*	CGY132	CAIQ
264	*Xanthomonas oryzae* pv. *oryzicola*	CGY133	CAIQ
265	*Xanthomonas oryzae* pv. *oryzicola*	CGY134	CAIQ
266	*Xanthomonas oryzae* pv. *oryzicola*	CGY135	CAIQ
267	*Xanthomonas oryzae* pv. *oryzicola*	CGY136	CAIQ
268	*Xanthomonas oryzae* pv. *oryzicola*	CGY138	CAIQ
269	*Xanthomonas oryzae* pv. *oryzicola*	CGY139	CAIQ
270	*Xanthomonas oryzae* pv. *oryzicola*	GXB1-6	CAIQ
271	*Xanthomonas oryzae* pv. *oryzicola*	GYC602	CAIQ
272	*Xanthomonas oryzae* pv. *oryzicola*	GYC608	CAIQ
273	*Xanthomonas oryzae* pv. *oryzicola*	GYC610	CAIQ
274	*Xanthomonas oryzae* pv. *oryzicola*	GYC612	CAIQ
275	*Xanthomonas oryzae* pv. *oryzicola*	GYC613	CAIQ
276	*Xanthomonas oryzae* pv. *oryzicola*	GYC614	CAIQ
277	*Xanthomonas oryzae* pv. *oryzicola*	GYC615	CAIQ
278	*Xanthomonas oryzae* pv. *oryzicola*	GYC616	CAIQ
279	*Xanthomonas oryzae* pv. *oryzicola*	GYC617	CAIQ
280	*Xanthomonas oryzae* pv. *oryzicola*	GYC620	CAIQ
281	*Xanthomonas oryzae* pv. *oryzicola*	GYC636	CAIQ
282	*Xanthomonas oryzae* pv. *oryzicola*	GYC638	CAIQ
283	*Xanthomonas oryzae* pv. *oryzicola*	GYC645	CAIQ
284	*Xanthomonas oryzae* pv. *oryzicola*	GYC646	CAIQ
285	*Xanthomonas oryzae* pv. *oryzicola*	GYC647	CAIQ
286	*Xanthomonas oryzae* pv. *oryzicola*	GYC652	CAIQ
287	*Xanthomonas oryzae* pv. *oryzicola*	GYC656	CAIQ
288	*Xanthomonas oryzae* pv. *oryzicola*	GYC659	CAIQ
289	*Xanthomonas oryzae* pv. *oryzicola*	GYC661	CAIQ
290	*Xanthomonas oryzae* pv. *oryzicola*	GYC663	CAIQ
291	*Xanthomonas oryzae* pv. *oryzicola*	GYC664	CAIQ
292	*Xanthomonas oryzae* pv. *oryzicola*	GYC665	CAIQ

No.	Species/Pathovar	Strain	Source
293	*Xanthomonas oryzae* pv. *oryzicola*	GYC669	CAIQ
294	*Xanthomonas oryzae* pv. *oryzicola*	GYC670	CAIQ
295	*Xanthomonas oryzae* pv. *oryzicola*	HANB1-25	CAIQ
296	*Xanthomonas oryzae* pv. *oryzicola*	HNB16-100	CAIQ
297	*Xanthomonas oryzae* pv. *oryzicola*	HNB16-97	CAIQ
298	*Xanthomonas oryzae* pv. *oryzicola*	IJB01-25	CAIQ
299	*Xanthomonas oryzae* pv. *oryzicola*	JSB3-28	CAIQ
300	*Xanthomonas oryzae* pv. *oryzicola*	RS105	CAIQ
301	*Xanthomonas oryzae* pv. *oryzicola*	RS60	CAIQ
302	*Xanthomonas oryzae* pv. *oryzicola*	SCBO-1	CAIQ
303	*Xanthomonas oryzae* pv. *oryzicola*	SCBO-7	CAIQ
304	*Xanthomonas oryzae* pv. *oryzicola*	SCBO-8	CAIQ
305	*Xanthomonas oryzae* pv. *oryzicola*	YNB1-3	CAIQ
306	*Xanthomonas oryzae* pv. *oryzicola*	YNB7-34	CAIQ
307	*Xanthomonas oryzae* pv. *oryzicola*	YNBO-1	CAIQ
308	*Xanthomonas oryzae* pv. *oryzicola*	YNBO-10	CAIQ
309	*Xanthomonas oryzae* pv. *oryzicola*	YNBO-11	CAIQ
310	*Xanthomonas oryzae* pv. *oryzicola*	YNBO-12	CAIQ
311	*Xanthomonas oryzae* pv. *oryzicola*	YNBO-13	CAIQ
312	*Xanthomonas oryzae* pv. *oryzicola*	YNBO-14	CAIQ
313	*Xanthomonas oryzae* pv. *oryzicola*	YNBO-15	CAIQ
314	*Xanthomonas oryzae* pv. *oryzicola*	YNBO-2	CAIQ
315	*Xanthomonas oryzae* pv. *oryzicola*	YNBO-4	CAIQ
316	*Xanthomonas oryzae* pv. *oryzicola*	YNBO-7	CAIQ
317	*Xanthomonas oryzae* pv. *oryzicola*	YNBO-8	CAIQ
318	*Xanthomonas oryzae* pv. *oryzicola*	ZJBO1-21	CAIQ
319	*Xanthomonas populi*	LMG 5743	LMG
320	*Xanthomonas populi*	NCPPB 2951	NCPPB
321	*Xanthomonas populi*	NCPPB 3038	NCPPB
322	*Xanthomonas sacchari*	LMG 471	LMG
323	*Xanthomonas sacchari*	NCPPB 4341	NCPPB
324	*Xanthomonas* sp. pv. *alangii*	LMG 470	LMG
325	*Xanthomonas translucens* pv. *arrhenatheri*	NCPPB 3229	NCPPB
326	*Xanthomonas translucens* pv. *arrhenatheri*	NCPPB 2699	NCPPB
327	*Xanthomonas vasicola* pv. *holcicola*	NCPPB 2417	NCPPB

ATCC, American Type Culture Collection

NCPPB, National Collection of Plant Pathogenic Bacteria

DSMZ, Deutsche Sammlung von Mikroorganismen und Zellkulturen

LMG, BCCM/LMG Bacteria Collection, Laboratory for Microbiology

ICMP, International Collection of Micro-organisms from Plants

CAIQ, Chinese Academy of Inspection and Quarantine
