# Supplementary material for: DNA Barcoding for Efficient Species- and Pathovar-Level Identification of the Quarantine Plant Pathogen Xanthomonas
Source: PLoS One. 2016 Nov 18;11(11):e0165995. doi: 10.1371/journal.pone.0165995 (PMC5115671; doi:10.1371/journal.pone.0165995)
Supplement: S2 Table — (PDF) [file pone.0165995.s002.pdf]

S2 Table. Additional *Xanthomonas* sequences obtained from GenBank

Species	Gene	No.[a]	Accession number
X. albilineans	*cpn60*	1	FP565176.1
X. arboricola pv. *juglandis*	*cpn60*	1	CP012251.1
X. axonopodis pv. *citri*	*cpn60*	13	AE008923.1;CP009028.1;CP009031.1;CP009034.1;CP009040.1;CP003778.1;CP009025.1;CP009019.1;CP009013.1;CP009007.1;CP009004.1;CP008992.1;CP004399.1
X. axonopodis pv. *citrumelo*	*cpn60*	1	CP002914.1
X. axonopodis pv. *dieffenbachiae*	*cpn60*	1	CP014347.1
X. axonopodis pv. *phaseoli*	*cpn60*	2	AF426387.1;AY922370.1
X. axonopodis pv. *vesicatoria*	*cpn60*	1	AM039952.1
X. campestris pv. *campestris*	*cpn60*	5	CP000050.1;AE008922.1;AM920689.1;CP012145.1;CP012146.1
X. campestris pv. *raphani*	*cpn60*	1	CP002789.1
X. fuscans subsp. *fuscans*	*cpn60*	1	FO681494.1
X. oryzae pv. *oryzae*	*cpn60*	5	CP013666.1;AE013598.1;AP008229.1;CP012947.1;CP007166.1
X. oryzae pv. *oryzicola*	*cpn60*	9	CP011955.1;CP011956.1;CP011957.1;CP011962.1;CP011958.1; CP011959.1; CP007221.1;CP011960.1;CP007810.1
X. sacchari	*cpn60*	1	CP010409.1
X. translucens pv. *undulosa*	*cpn60*	1	CP008714.1
X. arboricola	*avrBs2*	3	JQ387641.1；JQ425035.1；JQ387642.1
X. arboricola pv. *juglandis*	*avrBs2*	1	CP012251.1
X. axonopodis pv. *citri*	*avrBs2*	23	CP004399.1；AE008923.1；CP011827.2；CP003778.1；CP009037.1；CP009034.1；CP009031.1；CP009028.1；CP009025.1；CP009022.1；CP009019.1；CP009016.1；CP009013.1；CP009010.1；CP009007.1；CP009004.1；CP009001.1；CP008998.1；CP008995.1；CP008992.1；CP008989.1；CP006857.1；CP009040.1
X. axonopodis pv. *citrumelo*	*avrBs2*	1	CP002914.1
X. axonopodis pv. *dieffenbachiae*	*avrBs2*	1	CP014347.1
X. axonopodis pv. *vesicatoria*	*avrBs2*	2	AF114720.1； AM039952.1
X. campestris pv. *campestris*	*avrBs2*	6	CP012145.1；CP012146.1；CP011946.1；AM920689.1；AE008922.1；CP000050.1
X. fragariae	*avrBs2*	3	JQ387639.1；JQ387637.1；JQ387640.1
X. fuscans subsp. *fuscans*	*avrBs2*	1	FO681494.1
X. oryzae pv. *oryzae*	*avrBs2*	6	AP008229.1；CP000967.2；CP013666.1；CP012947.1AE013598.1；CP007166.1
X. oryzae pv. *oryzicola*	*avrBs2*	12	CP007810.1；CP003057.2；CP011955.1；CP011956.1；CP011957.1；CP011958.1；CP011959.1；CP011960.1；CP011961.1；CP011962.1；DQ641035.1；CP007221.1
X. albilineans	*gyrB*	5	EU498963.1；EU498985.1；EU267171.1；FP565176.1；EU499030.1
X. populi	*gyrB*	2	EU499016.1；EU499016.1
X. albilineans	16S rDNA	6	AB248373.1；AB248372.1；AB248371.1；AB248370.1；KU726266.1；NR_026316.1
X. populi	16S rDNA	1	NR 026320.1

[a] The number of the gene sequences obtained from GenBank.
